# Pathophysiology-Driven Anesthetic Management of Williams Syndrome for Combined Oral Surgery: A Case Report

**DOI:** 10.7759/cureus.115523

**Published:** 2026-08-31

**Authors:** Sherin Abdelhamid, Khaled Abuamra, Usama Abdelgwad, Aicha Idrissi, Gopinath Chowdappa, Cornelia Weidinger

**Affiliations:** 1 Department of Anesthesia, Dubai Hospital, Dubai Health, Dubai, ARE

**Keywords:** balanced anesthesia, dental rehabilitation, hemodynamic stability, pediatric anesthesia, perioperative management, ranula excision, supravalvular aortic stenosis, williams syndrome

## Abstract

Williams syndrome is a rare multisystem genetic disorder associated with developmental delay, characteristic dysmorphic features, congenital cardiovascular lesions, and increased perioperative anesthetic risk, particularly when supravalvular aortic stenosis or other vasculopathy is present. Children with Williams syndrome may also present practical perioperative challenges related to anxiety, poor cooperation, and potential airway difficulty, making individualized anesthetic planning essential. We report a 12-year-old boy with Williams syndrome who underwent ranula excision and complete oral rehabilitation under general anesthesia. Although preoperative cardiac evaluation revealed no severe structural anomalies, he was managed as high risk due to the inherent vulnerability of coronary perfusion in Williams syndrome. The perioperative strategy combined tight hemodynamic control with pharmacologic and nonpharmacologic measures to minimize sympathetic activation. This case highlights that apparently mild structural cardiac findings may not fully reflect perioperative risk in Williams syndrome and supports a pathophysiology-driven, individualized anesthetic approach even for seemingly minor noncardiac procedures.

## Introduction

Williams syndrome is a neurodevelopmental disorder caused by a microdeletion of chromosome 7q11.23 involving the elastin gene and is characterized by a distinctive facial appearance, cognitive and behavioral features, connective tissue abnormalities, and cardiovascular disease. Supravalvular aortic stenosis (SVAS) is the most common cardiovascular lesion, but diffuse arteriopathy and coronary abnormalities may also occur. Because SVAS creates a fixed left ventricular stroke volume, affected patients cannot effectively increase cardiac output to compensate for anesthesia-induced vasodilation. This physiological bottleneck makes them exquisitely vulnerable to even minor decreases in systemic vascular resistance; such drops can critically compromise diastolic coronary perfusion, rapidly precipitating perioperative myocardial ischemia or sudden cardiovascular collapse [1].

The anesthetic significance of Williams syndrome extends beyond the echocardiographic severity of a single lesion. Published series and reviews report increased rates of hemodynamic complications and describe anesthesia-related cardiac arrest in this population, supporting a cautious perioperative approach even for noncardiac surgery. In addition, developmental delay, anxiety, hypersociability, and craniofacial variation may complicate preoperative preparation and airway management [2].

This report describes the anesthetic management of a child with Williams syndrome who underwent combined ranula excision and complete oral rehabilitation, with particular emphasis on preoperative stress reduction, staged induction, hemodynamic control, and guided nasotracheal intubation, thereby underscoring evidence-based risk mitigation in a high-risk patient [3,4].

## Case presentation

Medical history

A 12-year-old boy weighing 37 kg and measuring 143 cm presented for elective sublingual ranula excision and complete oral rehabilitation under general anesthesia. Williams syndrome had been confirmed genetically by fluorescence in situ hybridization, which demonstrated a 7q11.23 microdeletion. The patient had been followed by multiple specialties, including genetics, cardiology, nephrology, otolaryngology, ophthalmology, and rehabilitation medicine. The patient had global developmental delay and speech delay. His additional medical history included failure to thrive during early childhood, bilateral hypermetropia, and bilateral epiphora that had resolved following probing and syringing.

The patient’s medical history included secondary arterial hypertension, which was well controlled between the 50th and 90th percentiles for his age with losartan 50 mg daily. An earlier trial of amlodipine had been discontinued secondary to gingival hypertrophy. Crucially, given the imperative to maintain adequate systemic vascular resistance and avert refractory post-induction hypotension in Williams syndrome, this angiotensin II receptor blocker (ARB) was intentionally withheld on the morning of surgery.

Preoperative comprehensive two-dimensional and Doppler echocardiography demonstrated normal cardiac situs, levocardia, and concordant atrioventricular and ventriculoarterial connections. The primary structural finding was a supra-aortic ridge causing mild SVAS, with a peak instantaneous pressure gradient of 24 mmHg. Additionally, the examination revealed mitral valve prolapse accompanied by mild mitral regurgitation. This regurgitant volume load resulted in mild left ventricular dilatation, characterized by a left ventricular end-diastolic dimension of 51 mm and an end-systolic dimension of 37 mm. Despite this localized dilatation, overall left ventricular systolic function remained preserved, demonstrating an ejection fraction of 55% and a fractional shortening of 28%. The proximal coronary arteries, aortic arch, pulmonary arteries, and systemic and pulmonary venous returns were structurally and morphologically normal. Both the interatrial and interventricular septa were intact, and only trivial pulmonary regurgitation was observed. Based on these findings, the cardiology service recommended routine one-year clinical follow-up (Figure 1).

**Figure 1:**
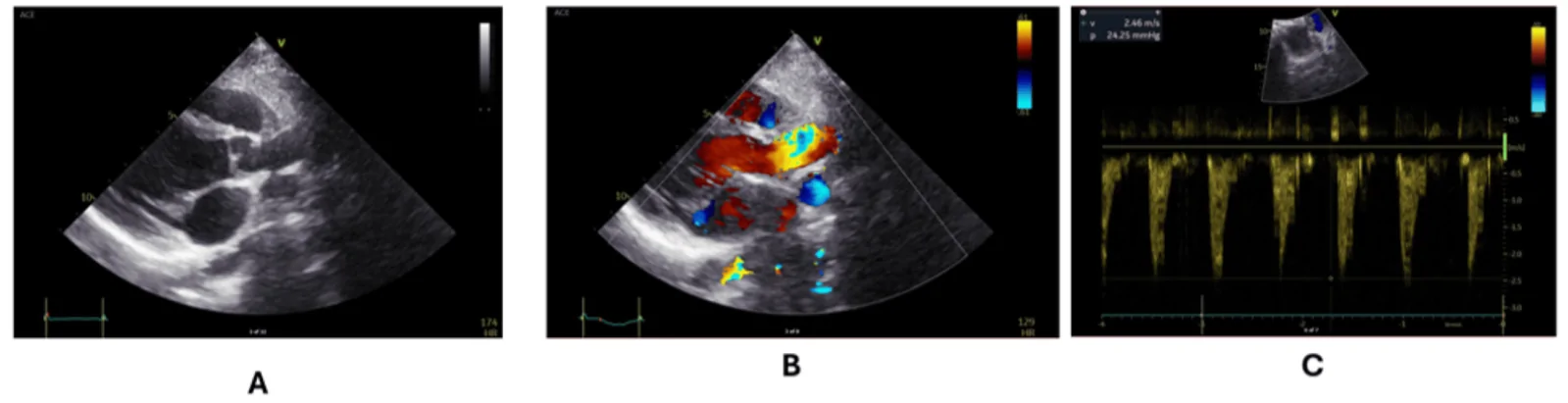
Echocardiographic demonstration of mild supravalvular aortic stenosis in Williams-Beuren syndrome. (A) Parasternal long-axis view showing focal narrowing at the sinotubular junction, consistent with supravalvular aortic stenosis. (B) Color Doppler image showing localized flow acceleration across the supravalvular aortic segment. (C) Continuous-wave Doppler tracing showing a peak velocity of 2.46 m/s, corresponding to a peak instantaneous pressure gradient of 24 mmHg, consistent with mild supravalvular aortic stenosis.

The preoperative electrocardiogram demonstrated sinus rhythm at 99 beats per minute with a normal frontal QRS axis, normal atrioventricular conduction, and no evidence of clinically significant conduction abnormalities or ischemic changes, alongside borderline QTc prolongation; the measured QTc was 452 ms (calculated using Bazett’s formula) (Figure 2). The proximal coronary arteries were visualized on echocardiography and reported as normal. Given the absence of ischemic symptoms and a baseline ECG free of ischemic changes, the cardiology team deemed advanced coronary imaging (such as CT angiography) unnecessary, thereby avoiding the inherent risks of procedural sedation. Nevertheless, anesthetic management proceeded under the assumption of potential occult microvascular vulnerability.

**Figure 2:**
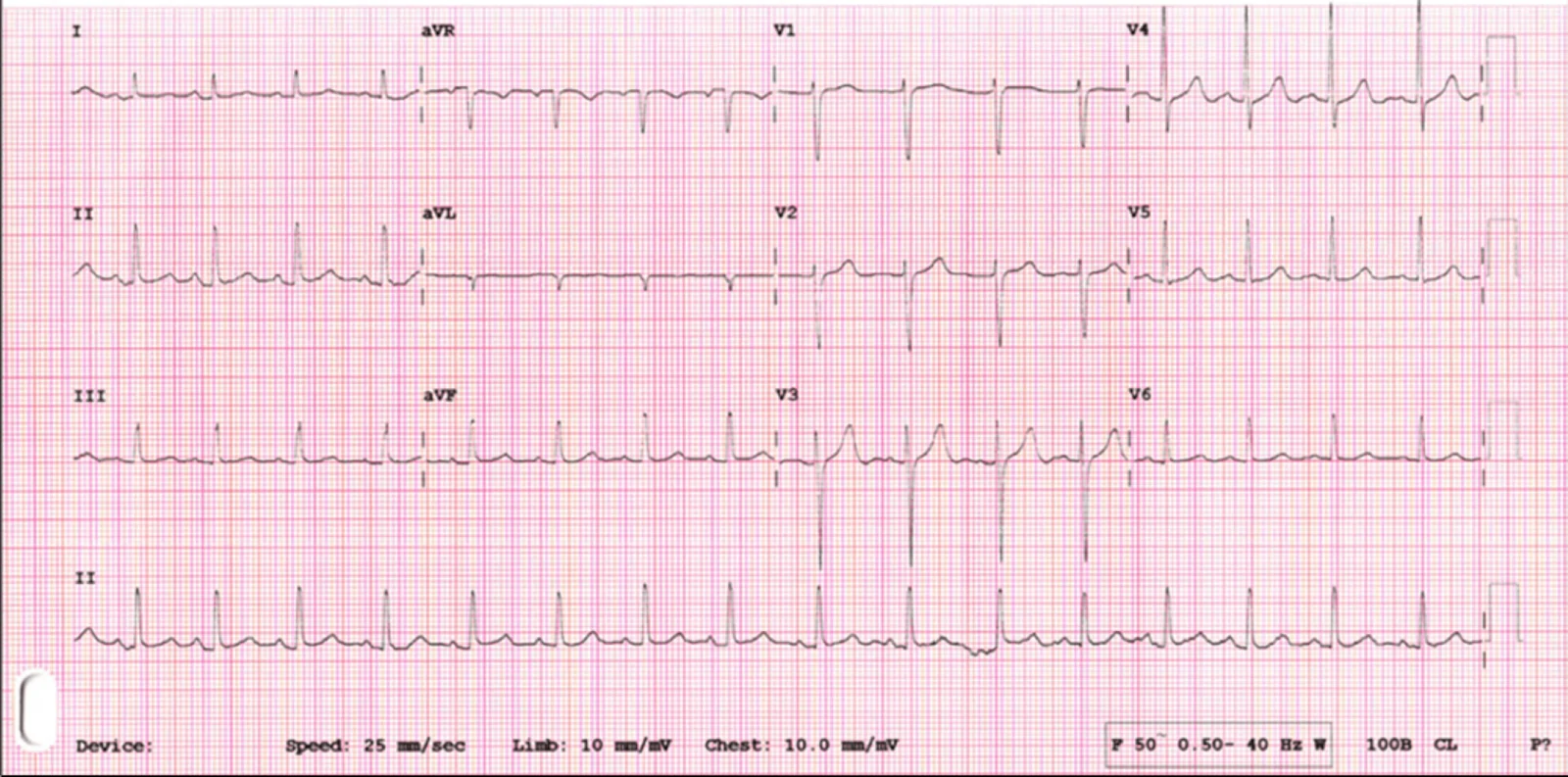
Preoperative 12-lead electrocardiogram. Sinus rhythm (99bpm) with normal frontal QRS axis, normal atrioventricular conduction, and no evidence of clinically significant conduction abnormalities or ischemic changes, QTc time measured QTc is 452 ms.

Renal assessment demonstrated asymmetric renal size, with a smaller left kidney. Dimercaptosuccinic acid (DMSA) scintigraphy showed differential renal function of 43% in the left kidney and 57% in the right kidney. Doppler ultrasonography and computed tomography angiography did not demonstrate hemodynamically significant renal artery stenosis. The patient remained under nephrology follow-up for blood-pressure optimization and renal protection.

The patient had previously undergone general anesthesia for adenoidectomy and bilateral probing and syringing for epiphora. Both anesthetics were uneventful, and no difficulty with oral intubation was documented.

Clinical examination

The patient exhibited craniofacial features characteristic of Williams syndrome, including microcephaly, a short nose with a broad tip, a high-arched palate, and widely spaced teeth. To objectively quantify the current airway risk, a formal preoperative airway assessment was conducted. Findings revealed an adequate mouth opening (interincisal gap >3.5 cm), a Mallampati Class II airway view, a thyromental distance >6 cm, and normal cervical spine mobility. These favorable objective metrics were consistent with the patient's documented history of two previous uneventful oral intubations. However, despite these reassuring findings, the combination of the syndromic high-arched palate, varied dentition, and the specific requirement for nasotracheal intubation prompted our team to anticipate potential difficulty with nasal tube advancement.

Preoperative vital signs were within normal limits for his age, including a blood pressure of 105/64 mmHg and a heart rate of 90 beats per minute. Preoperative laboratory investigations, including renal function tests, were unremarkable: serum creatinine was 0.5 mg/dL, phosphate was 4.1 mg/dL, uric acid was 4.6 mg/dL, magnesium was 2.8 mg/dL, serum calcium was 9.5 mg/dL, and potassium was 4.0 mEq/L. The patient had no known medication allergies.

Dental assessment identified a mixed dentition stage accompanied by poor oral hygiene. Clinical examination identified dental caries in permanent teeth #3, #14, and #19, as well as on the distal surface of primary tooth D. Additionally, an intact stainless-steel crown (SSC) was noted on primary tooth S. Consequently, the patient required excision of the sublingual ranula and complete oral rehabilitation, which specifically consisted of restorative dental fillings for the identified caries without the need for any extractions.

Anaesthetic management

Preoperative preparation emphasized anxiety reduction. Pharmacological premedication was intentionally avoided to prevent unmonitored hemodynamic fluctuations or respiratory depression. Instead, we relied entirely on a non-pharmacological anxiolysis strategy; a quiet environment was maintained, the mother was present during transfer and inhalational induction, and the child was allowed to choose his favorite music in the operating theatre reception area before entering the operating room. The same music was continued during induction and again during emergence, and no clinically significant sympathetic stress response was observed.

After transfer to the operating room, standard intraoperative monitoring was applied, including continuous electrocardiography (ECG) and non-invasive blood pressure (NIBP) monitoring. Anesthesia was initiated with a sevoflurane mask induction in the presence of the mother to facilitate intravenous (IV) cannulation, after which a 22-gauge catheter was inserted into the radial artery to enable continuous invasive blood pressure monitoring. Immediately following arterial line placement, a continuous IV phenylephrine infusion was initiated concurrently with IV induction to preemptively counteract any decline in systemic vascular resistance (SVR).

To achieve a hemodynamically stable transition and avoid the profound vasodilation often associated with standard high-dose single-agent inductions, we utilized a carefully titrated, balanced co-induction technique. This customized regimen included low-dose IV ketamine (total dose 15 mg; 0.4 mg/kg) to provide sympathetic support, IV midazolam (total dose 1 mg; 0.027 mg/kg) for enhanced anxiolysis, IV fentanyl (total dose 75 µg; 2.0 µg/kg) to effectively blunt the intubation reflex, and IV propofol (total dose 100 mg; 2.7 mg/kg) for hypnosis. By utilizing these agents synergistically at tailored doses, optimal depth of anesthesia was achieved while strictly preserving baseline hemodynamics. IV glycopyrronium (total dose 150 µg; 4.0 µg/kg) was administered immediately before intubation for its antisialagogue effect. Crucially, no neuromuscular blocking agent was utilized. The intentional preservation of spontaneous ventilation served to maintain negative intrathoracic pressure, thereby supporting venous return and cardiac preload, which is vital in SVAS, while also providing an added margin of airway safety. Despite the absence of paralysis, the synergistic deep plane of anesthesia achieved via fentanyl, propofol, and sevoflurane provided optimal intubating conditions with excellent jaw relaxation, facilitating an atraumatic first-pass guided nasotracheal intubation.

Anesthesia was subsequently maintained using a balanced technique comprising sevoflurane (1.0-1.5% inspired fraction) in an oxygen/air mixture and a continuous IV propofol infusion (4-6 mg/kg/h), with the depth of anesthesia continuously monitored via the bispectral index (BIS). For targeted sympatholysis and intraoperative opioid-sparing analgesia, a continuous IV dexmedetomidine infusion was administered at a rate of 0.25 µg/kg/h without a prior loading bolus, deliberately omitting the bolus to avoid acute bradycardia or transient hemodynamic swings and yielding a cumulative total dose of 12 µg (0.32 µg/kg) over the procedure.

Intraoperative hemodynamics were continuously supported by the phenylephrine infusion, maintained predominantly at 8 µg/min (approximately 0.22 µg/kg/min) and carefully titrated to effect based on invasive systemic blood pressure readings and BIS trends. Throughout the procedure, vital signs remained remarkably stable and closely mirrored baseline preoperative values. Invasive blood pressure was tightly maintained within a range of 95/60 mmHg to 115/75 mmHg. Heart rate was strictly controlled between 80 and 95 beats per minute without any episodes of unprovoked tachycardia or bradycardia. Oxygen saturation (SpO2) remained at 100%, and end-tidal carbon dioxide (EtCO2) was maintained between 35 and 40 mmHg.

Dexamethasone 0.1 mg/kg was administered intraoperatively for its multimodal benefits, including anti-inflammatory, analgesic-sparing, and antiemetic effects. Crucially, in light of the patient's borderline preoperative QTc prolongation, dexamethasone was specifically chosen for postoperative nausea and vomiting (PONV) prophylaxis to intentionally avoid 5-HT3 antagonists (e.g., ondansetron), which carry a known risk of further prolonging the QT interval, thereby contributing to both PONV prophylaxis and enhanced postoperative comfort.

Postoperative analgesia was managed via a tailored multimodal analgesic strategy. This included an intraoperative dexmedetomidine infusion (total dose 12 µg; 0.32 µg/kg) and intravenous paracetamol (15 mg/kg; 550 mg). Intraoperative fluid management was maintained using lactated Ringer's solution, which was deemed clinically appropriate given the patient’s normocalcaemic preoperative profile. A standard weight-based basal maintenance rate of 77 mL/hr was established. The patient had a preoperative fasting duration of 8 hours, resulting in an estimated fluid deficit of approximately 616 mL. Over the 75-minute surgical duration, fluid administration was carefully titrated to provide baseline maintenance and partially replace the fasting deficit, resulting in a total administered volume of 400 mL. Estimated blood loss was minimal at approximately 10 mL. Rather than a strictly “restricted” strategy, this represented a cautious, euvolemia-targeted approach; fluid administration was strictly controlled to replace true deficits and ensure adequate cardiac preload while deliberately avoiding aggressive empirical fluid boluses to prevent volume overload and subsequent hypertensive episodes in the context of Williams syndrome.

Airway management was performed using a cuffed, soft nasal Ring-Adair-Elwyn (RAE) endotracheal tube with an internal diameter of 5.5 mm. Given the patient’s craniofacial dysmorphism and the high suspicion for a difficult nasal intubation, a flexible, 8-French (Fr) nasogastric tube was utilized as an internal guide catheter through the endotracheal tube prior to insertion. This technique served to soften the leading edge and minimize mucosal trauma during advancement. The tube was gently advanced through the right naris. Video laryngoscopy was performed using a GlideScope®, yielding a Cormack-Lehane grade I view of the glottic opening. Tracheal intubation was completed uneventfully on the first attempt. The tube was secured at a depth of 19 cm at the right naris. Correct intratracheal placement was immediately confirmed by the presence of equal bilateral breath sounds upon auscultation and a consistent square-wave capnography (EtCO2) tracing. The intubation proceeded smoothly without any epistaxis, mucosal bleeding, or other airway complications. Ventilation was initially provided in volume-controlled SIMV/assisted flow mode (Draeger Zeus), with a gradual transition to spontaneous ventilation as the procedure progressed (Figure 3).

**Figure 3:**
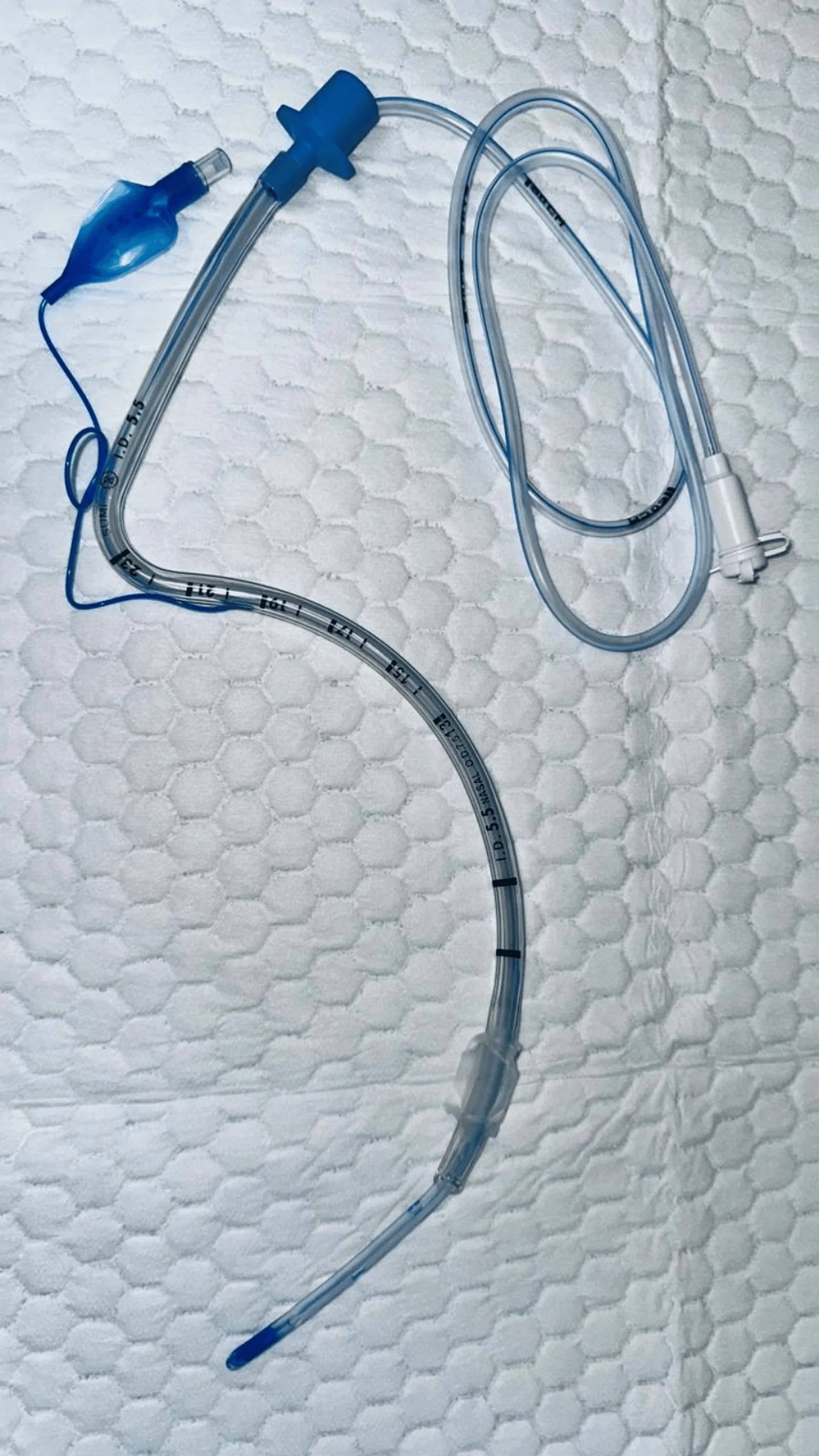
Preformed nasal Ring–Adair–Elwyn (RAE) endotracheal tube with a nasogastric tube used as a guiding device to facilitate atraumatic nasotracheal intubation.

Emergence and recovery were conducted in a low-stimulation environment with continuation of the child’s preferred music. Postoperative observation in the post-anesthesia care unit lasted 35 minutes, during which the patient remained cardiovascularly stable, pain-free, fully conscious, and comfortable on room air. No additional postoperative analgesia was required.

Given the patient's underlying Williams syndrome and the associated cardiovascular risks, the initial care plan mandated 24 hours of continuous inpatient postoperative monitoring. However, at six hours postoperatively, the patient was remarkably active and hemodynamically stable, achieving a maximum score of 10 on Chung's Modified Post-Anesthetic Discharge Scoring System (Modified PADSS). This score confirmed stable vital signs, steady ambulation, adequate pain control, and the absence of postoperative nausea. Following a thorough clinical assessment verifying that all objective discharge criteria were strictly met and after confirming six hours of completely stable continuous ECG and blood pressure monitoring, a shared decision was made by the cardiology team to accommodate the mother's strong request for early discharge. Telephonic follow-up on postoperative day one revealed a smooth recovery at home with no clinical concerns.

## Discussion

This case underscores the critical necessity of individualized anesthetic planning for pediatric patients with Williams syndrome. Although preoperative echocardiography in this patient demonstrated only mild SVAS with preserved ventricular systolic function, such isolated hemodynamic measurements can be deceptively reassuring. The underlying pathophysiology of Williams syndrome is characterized by a diffuse elastin arteriopathy, which frequently manifests as occult coronary artery abnormalities, altered vascular compliance, chronic systemic hypertension, and electrocardiographic aberrations [1,2]. Consequently, the true perioperative cardiovascular vulnerability of these patients, specifically the risk of myocardial ischemia and biventricular strain, cannot be reliably predicted by the severity of a single echocardiographic obstructive lesion [2].

The clinical significance of this diffuse cardiovascular pathology cannot be overstated; sudden, often fatal intraoperative deterioration has been extensively documented in patients with Williams syndrome, even during minor procedures [3]. To mitigate this severe risk, our anesthetic strategy was strictly tailored to optimize the myocardial oxygen supply and demand balance. The primary physiological goals prioritized the maintenance of adequate cardiac preload and SVR, alongside the preservation of normal sinus rhythm to ensure sufficient diastolic coronary perfusion. The early placement of an invasive arterial catheter was pivotal to this strategy, enabling continuous, beat-to-beat hemodynamic surveillance and facilitating the prompt, precise titration of vasoactive agents at the first sign of instability [3,4].

Ultimately, the anesthetic management of this case underscores the inherent intricacies of syndromic pediatric anesthesia. Performing concurrent procedures, specifically a sublingual ranula excision and restorative dental rehabilitation, in a patient with genetically confirmed Williams syndrome, secondary hypertension, mitral valve prolapse, and asymmetric renal function required meticulous multidisciplinary coordination. Crucially, this case demonstrates that a highly vigilant approach is necessary to anticipate systemic cardiovascular complications, emphasizing that a seemingly benign preoperative echocardiogram does not preclude severe perioperative risk [5].

The patient had anxiety and hyperacusis consistent with the behavioral phenotype associated with Williams syndrome [6,7]. These features were considered relevant to preoperative cooperation, induction, and emergence. His mother was the primary caregiver and was present during preoperative preparation, transfer, inhalational induction, and emergence. No pharmacological premedication was administered. In patients with Williams syndrome, sedative premedication (e.g., oral midazolam) carries a significant risk of unmonitored hemodynamic instability or respiratory depression. Instead, we relied entirely on non-pharmacological anxiolysis, specifically a low-stimulation environment, maternal presence, and patient-selected music, which successfully maintained baseline hemodynamics prior to induction.

The perioperative management of patients with Williams syndrome is fraught with significant cardiovascular risks. The hallmark cardiac lesion in these patients is SVAS, which can lead to biventricular hypertrophy and severely compromised coronary perfusion. Sudden cardiac arrest during anesthesia induction and maintenance is a well-documented and tragic complication in this population [4,8]. Consequently, the primary hemodynamic goals are to maintain SVR, ensure adequate preload, and strictly avoid tachycardia or myocardial depression.

To achieve these goals, continuous invasive hemodynamic monitoring is essential. A balanced co-induction technique was meticulously utilized. While standard induction doses of ketamine are traditionally controversial in patients with coronary artery disease due to the risk of tachycardia and increased myocardial oxygen demand, its use in this context was highly modified. We utilized a low adjunct dose of ketamine (0.4 mg/kg) specifically for its sympathomimetic properties to preserve SVR and counterbalance the potentially fatal vasodilatory and myocardial-depressant effects of propofol. To mitigate the risk of ketamine-induced tachycardia, it was synergistically co-administered with fentanyl and an ongoing dexmedetomidine infusion. This tailored pharmacological combination successfully maintained diastolic coronary perfusion pressure without precipitating chronotropic stress [9,10]. The cornerstone of intraoperative hemodynamic support in patients with SVAS is the meticulous maintenance of diastolic blood pressure to guarantee coronary perfusion. Any sudden decrease in SVR, combined with the fixed stroke volume characteristic of SVAS, can precipitously induce myocardial ischemia. We utilized a continuous phenylephrine infusion (predominantly at 0.22 µg/kg/min) because its pure alpha-1 adrenergic agonism effectively maintains SVR. Phenylephrine was chosen to support SVR while limiting increases in heart rate. Agents associated with substantial chronotropic stimulation, such as ephedrine, were deliberately avoided because tachycardia may be poorly tolerated in patients with SVAS and possible coronary involvement [1,4,11]. Crucially, the infusion was meticulously titrated to match the patient’s baseline blood pressure, thereby optimizing coronary blood flow while strictly avoiding excessive left ventricular afterload that could precipitate myocardial ischemia.

Furthermore, anesthesia was maintained via a balanced technique utilizing a propofol infusion, sevoflurane, and dexmedetomidine, guided by bispectral index (BIS) monitoring. This multimodal combination allowed for lower fractional dosing of each hypnotic agent, minimizing direct dose-dependent myocardial depression, while the cumulative vasodilatory effect was effectively counteracted by the continuous phenylephrine infusion. The addition of dexmedetomidine provided profound sympatholysis, blunting tachycardic responses to surgical stimulation while maintaining hemodynamic stability. Finally, these combined strategies culminated in a highly controlled emergence. The integration of dexmedetomidine, dexamethasone, and paracetamol provided a robust multimodal, opioid-sparing analgesic pathway, successfully ensuring an emergence devoid of pain-induced sympathetic surges. Independently, the intentional avoidance of neuromuscular blocking agents precluded the need for pharmacological reversal, thereby eliminating the risk of reversal-induced hemodynamic fluctuations and further securing cardiovascular stability during extubation [5,8,10].

A multimodal analgesic strategy was employed, utilizing an intraoperative dexmedetomidine infusion (total dose 12 µg; 0.32 µg/kg) alongside intravenous paracetamol (15 mg/kg; 550 mg). The incorporation of dexmedetomidine provided an effective opioid-sparing analgesic effect while simultaneously offering beneficial sympatholysis, which was critical for maintaining hemodynamic stability given the patient's Williams syndrome and baseline hypertension. To maintain a nephroprotective strategy in the context of the patient's Williams syndrome history, nonsteroidal anti-inflammatory drugs were withheld.

Intraoperative fluid management was maintained utilizing lactated Ringer's solution. Although Williams syndrome can be associated with idiopathic hypercalcemia, the patient’s preoperative serum calcium levels were confirmed to be within normal limits, making the use of this balanced, calcium-containing crystalloid safe and appropriate. Fluid administration was carefully titrated to maintain adequate ventricular preload while strictly avoiding volume overload in the setting of the patient's baseline hypertension [1,3].

Airway management in pediatric patients with Williams syndrome presents distinct challenges, primarily due to inherent craniofacial dysmorphism, which often includes maxillary hypoplasia, a depressed nasal bridge, and micrognathia. These anatomical variations significantly increase the risk of difficult airway management and mucosal trauma during nasotracheal intubation [1,3]. Epistaxis is a particularly hazardous complication in pediatric anesthesia, as blood can rapidly obscure the laryngoscopic view and increase the risk of pulmonary aspiration. To mitigate this, we utilized a catheter-guided insertion technique. As demonstrated in this case, passing a flexible 8-French nasogastric tube through the endotracheal tube acts as an internal stent, effectively blunting the sharp leading bevel of the nasal RAE tube. This technique allows the tube to glide atraumatically along the nasal floor and past the turbinates, a method well documented to significantly reduce the incidence of epistaxis [12]. Furthermore, our atraumatic first-pass success was facilitated by the preemptive use of video laryngoscopy (GlideScope®), which is highly recommended in syndromic pediatric airways to provide a superior glottic view without requiring traditional alignment of the airway axes.

Behavioral management was another important component. Anxiety and hyperacusis may increase sympathetic stimulation and make induction and emergence more difficult. Parental presence, a low-stimulation environment, and patient-selected music were used throughout the perioperative period. Same-day discharge was successful in this patient following an uncomplicated recovery. This outcome should not be interpreted as evidence that ambulatory anesthesia is universally appropriate for Williams syndrome. Published experience demonstrates that serious cardiovascular events can occur during or after anesthesia, and patient selection, contemporary cardiovascular assessment, institutional capability, and postoperative observation remain essential [11,13].

## Conclusions

Anesthetic management of children with Williams syndrome should account for the possibility of diffuse vascular and coronary disease even when echocardiography demonstrates only mild SVAS. A structured plan emphasizing cardiovascular assessment, avoidance of abrupt hemodynamic changes, readiness for vasoactive support, anticipation of airway challenges, and individualized anxiety reduction may facilitate safe anesthesia for selected noncardiac procedures. This case supports a pathophysiology-driven approach rather than reliance on the severity of a single echocardiographic lesion when planning anesthesia for children with Williams syndrome.
